# Measurement Uncertainty Impacts Diagnosis of Diabetes Mellitus: Reliable Minimal Difference of Plasma Glucose Results

**DOI:** 10.1007/s13300-019-00740-w

**Published:** 2019-12-16

**Authors:** Sandra Keutmann, Stephanie Zylla, Mathilde Dahl, Nele Friedrich, Rüdiger Landgraf, Lutz Heinemann, Anders Kallner, Matthias Nauck, Astrid Petersmann

**Affiliations:** 1 Institute of Clinical Chemistry and Laboratory Medicine, University Medicine Greifswald, Greifswald, Germany; 2 DZHK (German Centre for Cardiovascular Research), Partner Site Greifswald, University Medicine Greifswald, Greifswald, Germany; 3 German Diabetes Foundation (DDS), Munich, Germany; 4 Science-Consulting in Diabetes GmbH, Neuss, Germany; 5 Department of Clinical Chemistry, Karolinska University Hospital, Stockholm, Sweden; 6 Present Address: Institute of Clinical Chemistry, University Medicine Göttingen, Göttingen, Germany

**Keywords:** Assay performance, Coefficient of variation, Imprecision, Internal quality control, Minimal difference, Rili-BAEK, Westgard rules

## Abstract

**Introduction:**

The diagnosis of diabetes mellitus is based on suitable cut-off values of specific biomarkers, such as the concentration of glucose in plasma. The German Diabetes Association has very recently published a clinical practice guideline on the definition, classification and diagnosis of diabetes mellitus that recommends measurements of plasma glucose concentration have an imprecision defined as a minimal difference (MD) of at a fasting plasma glucose concentration of 7.0 mmol/L. To obtain reliable values for the MD, we investigated long-term and short-term measurement uncertainty.

**Methods:**

The imprecision was determined by two approaches: (1) a long-term dataset with imprecision based on the Guideline of the German Medical Association on Quality Assurance in Medical Laboratory Examinations (Rili-BAEK), in a medical laboratory operating 24/7, using internal quality control (IQC) data for four concentrations during a 10-year period; and (2) a detailed short-term dataset with imprecision assessed by hourly measurements of control materials. These datasets were used to calculate the MD cut-off (MD_cut-off_) as: ${\text{MD}}_{\text{cut-off}} = k \times {\text{SD}}$  = 2  $\times {\text{SD}}$, where SD is the standard deviation and* k* = 2 represents a confidence level of 95%.

**Results:**

The MD_cut-off_ of ≤ 0.7 mmol/L at a fasting plasma glucose concentration of 7.0 mmol/L (MD_cut-off 7.0_) for the long-term and the short-term approaches were 0.44 and 0.40 mmol/L, respectively. The MD_cut-off 7.0_ from both approaches was therefore below the recommended value of 0.7 mmol/L. It was noted that the variability in performance within and between instruments can be covered by reporting the long-term MD_cut-off 7.0_ across all connected instruments. In this study, stable results for the MD_cut-off 7.0_ were obtained after 1 year.

**Conclusion:**

Imprecision as measured by IQC data is remarkably stable over many years of operation. Current imprecision assessment usually focuses on only single instruments, whereas clinicians perceive the measurement as the result of the combined analytical performance of all instruments used for a certain assay. In the clinical setting, the MD may be a more useful measure of imprecision, and we suggest deriving the MD_cut-off_ combined from all instruments and control cycles that are used in the patient care setting for a given analyte.

**Electronic supplementary material:**

The online version of this article (10.1007/s13300-019-00740-w) contains supplementary material, which is available to authorized users.

## Key Summary Points

**Table:** 

All laboratory measurements are subject to measurement uncertainty, which can be described using the coefficient of variation (%CV). Despite the pervasive presence of high measurement uncertainty and the enormous impact it may have on the diagnosis of diabetes mellitus (DM), measurement uncertainty has not received appropriate attention by clinical users.
Our aim was to create awareness of the fact that the magnitude of measurement uncertainty especially impacts the diagnosis of DM when cut-off values are used. We calculated the recommended “minimal difference (MD)”, a value recently developed for this purpose, based on detailed short-term and long-term (10 years) internal quality control data for state-of-the-art glucose concentration measurements.
The MD represents the smallest difference between two values that is statistically significant, taking the specific measurement uncertainty into account; it is based on the standard deviation and expressed in the unit of the measurand to enable easy use by clinicians.
At the fasting plasma glucose cut-off of 7.0 mmol/L (126 mg/dL), which represents a frequently used cut-off value for the diagnosis of DM, the MD cut-off (MD_cut-off 7.0_) for the investigated assay was 0.44 mmol/L and remained below the recommended maximum MD of 0.7 mmol/L.
State-of-the-art glucose concentration measurements with low imprecision are associated with a considerable MD and therefore may impact cut-off points for diagnosing diabetes. It is important to note that imprecisions allowed for by guidelines or legal requirements are considerably higher than those observed in this study. Consequently, imprecision in glucose assays should also be reported as MD to enable clinical users to consider imprecision when relying on the results of a glucose assay to diagnose DM.

## Introduction

The diagnosis of diabetes mellitus (DM) is based on suitable cut-off values for specific biomarkers, such as the plasma glucose concentration. The World Health Organization (WHO), American Diabetes Association (ADA) and German Diabetes Association (DDG) recommend assaying samples of venous plasma, with a cut-off for fasting plasma glucose (FPG) of ≥ 7.0 mmol/L (≥ 126 mg/dL) and for 2-h plasma glucose of ≥ 11.1 mmol/L (200 mg/dL) after ingestion of 75 g of glucose in the oral glucose tolerance test [1–3]. According to WHO, impaired fasting glucose (IFG) is diagnosed when the plasma glucose level is between 6.1 and 6.9 mmol/L (110–125 mg/dL); according to ADA and DDG, the interval in plasma glucose level for IFG diagnosis is 5.6–6.9 mmol/L (100–125 mg/dL) [3].

### A Laboratory Result is Complete only when Reported Together with the Associated Measurement Uncertainty

Laboratory reports are often treated like a bank account statement: the value that is given on the statement is understood to be the actual amount of money in the bank account, and deviation by a few cents more or less is not acceptable. In contrast to the values on bank account statements, however, laboratory measurements are always subject to measurement uncertainty, and a few “cents” more or less in the results may represent the same value, depending on the magnitude of the associated uncertainty. Although it is generally accepted that a measurement result is only complete if the associated uncertainty is reported together with the measurement result, as stated in “The Guide to the Expression of Uncertainty in Measurements” [4], the uncertainty is usually not reported to the clinical end-user. Medical laboratories commonly report imprecision as the coefficient of variation (%CV) [5]. The %CV, as given in Eq. (1), may be difficult to immediately translate to a difference between two consecutive results or between a result and a cut-off value. It is important that the end-user—the clinician—takes the measurement uncertainty into account when comparing a laboratory result to a recommended cut-off value, such as, for example, when diagnosing or treating DM. Therefore, the uncertainty expressed in the units of the measurand, i.e. the minimal difference (MD), would be a more useful metric.

### Description of Minimal Difference

The DDG recently published a clinical practice guideline on the definition, classification and diagnosis of DM that recommends minimum requirements for the imprecision of glucose concentration measurements, expressed as the MD [2]. The MD is not a novel metric and has been described previously—for example, in the approved guideline of the Clinical and Laboratory Standards Institute (CLSI) EP29-A on “Expression of Measurement Uncertainty in Laboratory Medicine” [6]. The MD represents the smallest difference between two values that is statistically significant, taking the specific measurement uncertainty into account. It is based on the standard deviation (SD) and is expressed in the unit of the measurand so that clinicians are able to apply it directly to the result [7].

**Fig. 1:**
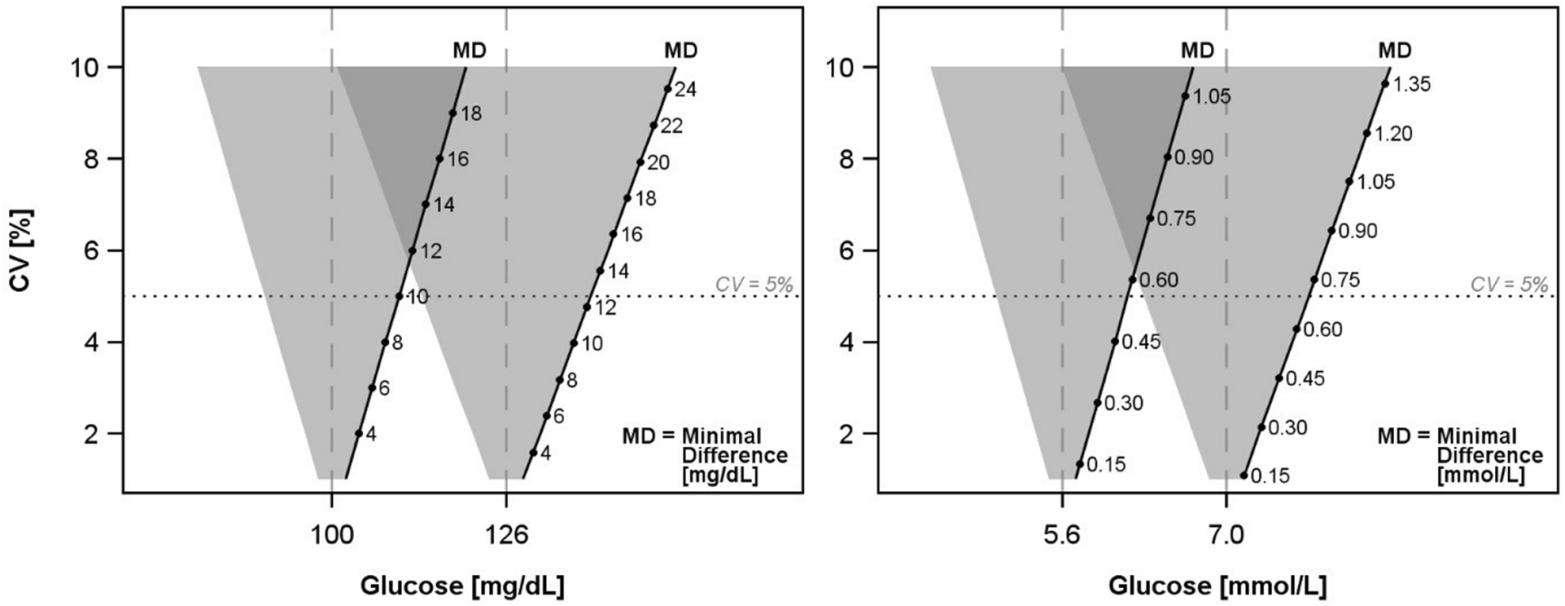
Relation between coefficient of variation (*%CV*) and minimal difference (*MD*) at glucose concentration cut-offs used for the diagnosis of diabetes mellitus. The width of the funnels at each %CV is the MD and can be read on the oblique scale. The vertical dotted lines are the cut-off values. The overlapping areas above the intersection of the funnels correspond to %CVs where the difference between 5.6 and 7.0 mmol/L glucose cannot be ascertained. Figure is reproduced with permission from M Nauck, A Petersmann, D Müller-Wieland, et al. Definition, Klassifikation und Diagnostik des Diabetes mellitus. Diabetol Stoffwech. 2018;13

At the FPG cut-off of 7.0 mmol/L, the DDG recommends a MD cut-off (MD_cut-off_) of ≤ 0.7 mmol/L (MD_cut-off 7.0_) for FPG measurements used for the diagnosis of DM. At this glucose concentration of 7.0 mmol/L, a SD = 0.35 mmol/L and a MD of 0.7 mmol/L (Eq. 5) correspond to a %CV of ≤ 5%. A %CV of 6% would correspond to 0.84 mmol/L, i.e. any value between 6.16 and 7.84. Applied to a concentration of 5.6 mmol/L the interval would be 4.93–6.27 mmol/L. An analytical %CV of this magnitude, or larger, would therefore not differentiate between diabetes and non-diabetes. This is illustrated by the intersection and overlap of the “funnels” in Fig. 1.

Current quality assurance regulations, such as the commonly practiced Westgard control rules [8] or the compulsory Guideline of the German Medical Association on Quality Assurance in Medical Laboratory Examinations (Rili-BAEK) [9], usually require at least two internal quality control (IQC) samples of different concentrations per assay, applied at least once each per instrument per day. If the daily requirements of the control system are met, no further action need be taken and the results are released. It is thus assumed that results obtained between two accepted control samples meet the quality criteria [5].

Here we report our assessment of the analytical imprecision of glucose concentration measurements on a continuously operated state-of-the-art measurement system. Glucose measurements were performed on three individual instruments connected to an automatic random sample distribution system. Two approaches were chosen: (1) long-term imprecision calculated from IQC data obtained in a regular patient care setting between 2009 and 2018; and (2) detailed short-term imprecision assessed hourly through the measurement of additional control materials during a 1-week period. The MD_cut-off_ of both approaches was calculated and compared for the FPG cut-off value of 7.0 mmol/L (126 mg/dL) for the diagnosis of DM.

The aim of the study was to provide reliable imprecision data for glucose concentration measurements and translate this imprecision into a clinical useable term, namely the MD. This information is important in view of the fact that each measurement result is subject to imprecision, and clinicians should be aware of the magnitude of this imprecision, especially when using cut-offs for diagnostic purposes, such as in the diagnosis of DM.

## Methods

All measurements were conducted at the Institute of Clinical Chemistry and Laboratory Medicine of the University Medicine of Greifswald, which operates 24 h, 7 days a week. The long-term study was performed between January 2009 and December 2018. The measuring systems were used for patient care and complied with the Rili-BAEK guideline. The plasma glucose concentrations were measured using calibrators, reagents and instruments (hexokinase-glucose-6-phosphate dehydrogenase method [10]) on three Dimension Vista 1500 (instruments 1–3) that were connected by a laboratory automation system (all from Siemens Healthcare GmbH, Eschborn, Germany). The automation system was changed from StreamLab to FlexLab (instrument 4; Siemens Healthcare GmbH) in 2012. Both automation systems distributed the samples to analytical Dimension Vista instruments 1–3. The IQC material Tru Liquid Monitrol (Thermo Fisher Scientific, Schwerte, Germany) was used from 2009 until December 2013, after which time it was changed to Liquid Assayed Multiqual (Bio-Rad Laboratories, Munich, Germany).

No patient material was used for this study; therefore, the study did not need to be registered, and it did not require the approval of an institutional ethics committee.

### Minimal Difference

The MD is based on the SD and is expressed in the unit of the measurand to enable clinicians to apply it directly to the result [7]. If a coverage factor (*k*) of 2 is used, the MD corresponds to a confidence level of about 95%. If two consecutive measurements are compared, the measurement uncertainty of both must be considered (Eq. 2). If measurement uncertainty is the same for each measurement, the equation can be simplified, as given in Eq. (3). If a measurement result is compared to a fixed cut-off value that is normally considered to be devoid of an uncertainty, the MD can be simplified to the MD_cut-off_ as calculated by Eq. (4), which in turn can be simplified to Eq. (5).

1 $$ {\text{\% CV}} = \frac{\text{SD}}{{\bar{X}}} \times 100;\; {\text{SD}} = \frac{{\bar{X} \times \% CV}}{100} $$

2 $$ {\text{MD}} = k \times \sqrt {{\text{SD}}_{1}^{2} + {\text{SD}}_{2}^{2} } $$

If SD_1_ = SD_2_:

3 $$ {\text{MD}} = k \times \sqrt {2 \times {\text{SD}}^{2} } = 2 \times {\text{SD}} \times \sqrt 2 $$

If SD_2_ = 0:

4 $$ {\text{MD}}_{\text{cut-off}} = k \times \sqrt {{\text{SD}}_{1}^{2} }, $$

which can be simplified to:

5 $$ {\text{MD}}_{\text{cut-off}} = k \times {\text{SD}} = 2 $$

In Eq. (1) to (5), $\bar{X}$ is the mean and *k* = 2, which represents a confidence level of 95%.

### Long-Term Experiment

#### Imprecision

Documented IQC data from the laboratory information system (LIS) were used retrospectively for the study period of 10 years. Control cycles were evaluated on a monthly basis, separately for each instrument, IQC level and much of the control material. Cycles with fewer than 15 IQC results were excluded.

### Short-Term Experiment

#### Workload

There was a total of 2016 possible measurement time points for all four instruments. The actual number of measurements was documented in order to correctly handle interruptions for service and maintenance. The overall workload, i.e. the total number of measurements per hour of the day, was retrieved retrospectively from the LIS for each of the instruments.

#### Imprecision

Control material was measured hourly over a period of 1 week in September 2012. Samples were directly introduced into each Dimension Vista instrument (1–3) or randomly through FlexLab, imitating the general work flow of patient samples. The latter procedure represents instrument 4. Performance of the instruments and the measurements were independently monitored with separate IQC materials according to the rules in the Rili-BAEK. Acceptance of these rules was the inclusion criterion for the measurements of both the patient care and study samples. Short-term imprecision results were performed as if they were patient samples and collected from the LIS retrospectively; they were not reported separately, nor used in the Rili-BAEK-based IQC system. Short-term control cycles were evaluated on a daily basis from the hourly measurements, separately for each instrument, IQC level and lot number.

Three levels of control material with concentrations of about 3, 7 and 20 mmol/L (Bio-Rad Liquid Assayed Multiqual; lot no. 45,631-1 through -3; Bio-Rad Laboratories) were used. For each level, a sufficient volume of each material to cover the needs for 1 day was thawed and pooled for each concentration and then aliquoted into barcode-labeled and capped tubes suitable for use in the instruments. The aliquoted material was stored at 2–5 °C until use (maximum storage time 24 h).

### Calculations and Statistics

Calculation of the %CV and MD, including descriptive statistics and boxplots, was performed using R (version 3.5.0; release 23 March 2018; https://www.r-project.org/). Statistical differences between instruments and years were calculated using analysis of variance, and Tukey’s HSD test was used as the post hoc test. Frequency graphs were designed using SAS version 9.4 software (SAS Institute Inc., Cary, NC, USA). The MD was calculated using Eq. (5), i.e. under the assumption that the cut-off was not liable to an uncertainty. All calculations are given in Electronic Supplementary Material (ESM) S1.

In the long-term approach, the MDs were calculated monthly for each instrument using the IQC results and then cumulated as a moving average to obtain a reliable value [11]. In the short-term approach, the MDs were calculated daily for each instrument. Equations (1) and (5) were applied for both approaches. All MDs were summarized in boxplots for the whole study period and also reported in detail by years and instruments, respectively.

The 95th percentiles of the MD_cut-off_ distributions were used to obtain reliable values for the MD suitable for the diagnosis of DM. The MD_cut-off_ from the different concentrations tested in this study were used to calculate a linear equation that would allow the MD_cut-off_ for other concentrations, such as the MD_cut-off 7.0_, to be estimated.

Since the material used in the short-term experiment was pooled and then distributed to the instruments, an evaluation of the bias among the instruments was possible. This bias was calculated separately for each instrument by subtracting the mean MD_cut-off_ of each instrument from the median MD_cut-off_ of all instruments.

#### Compliance with Ethics Guidelines

This article does not contain any studies with human participants or animals performed by any of the authors.

## Results

### Long-Term Experiment

#### Imprecision

**Fig. 2:**
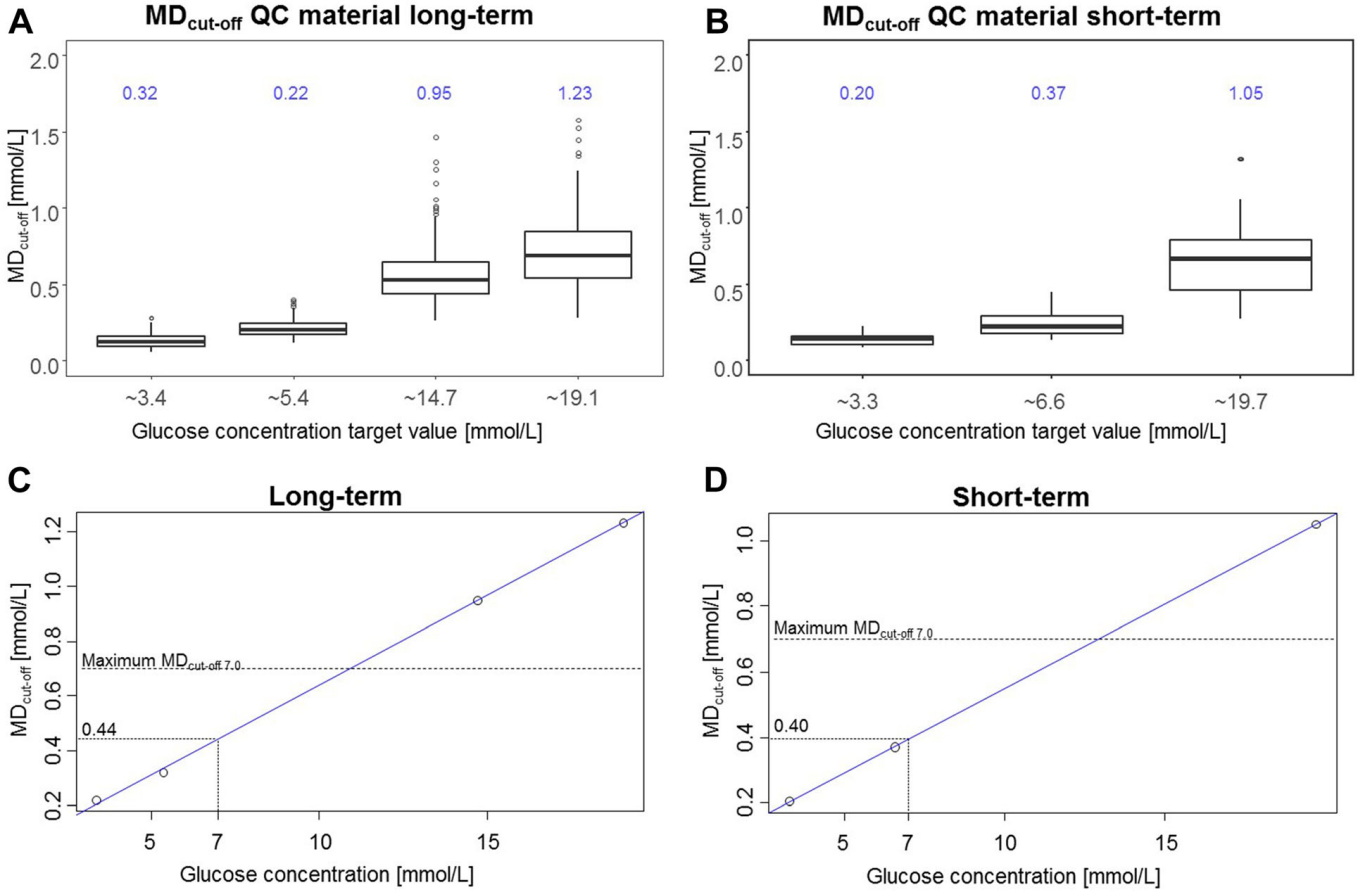
Distribution of the MD cut-offs (MD_cut-off_) at different concentrations of glucose (**a**, **b**) and linear regressions (**c**, **d**) based on the 95th percentile MD_cut-off_ (values are given above the boxplots in **a** and **b**). The linear regression (blue solid line) can be used to derive the MD at a plasma glucose cut-off value of 7.0 mmol/L (*MD*_*cut-off 7.0*_) as indicated by the solid black line. The maximum MD_cut-off 7.0_ according to the German Diabetes Association (DDG) is marked by the horizontal dotted line.** a**,** c** Long-term internal quality control (IQC) data from 2009 to 2018,** b**,** d** short-term data from hourly measurements of QC material during 1 week

Long-term glucose measurement imprecision was calculated from the IQC samples tested during patient care under routine conditions. The calculated values converged and reached a stable level after 30 observations. All results were obtained following the Rili-BAEK and are summarized in Fig. 2. The MD_cut-off 7.0_ was calculated from the linear equation *y* = 0.065*x −* 0.013, which was derived from four concentrations (3.2, 5.5, 15.9, and 19.2 mmol/L) and determined to be 0.44 mmol/L (Fig. 2c). The MD_cut-off 7.0_ differed marginally but significantly between the instruments (ESM S2, S3). This tendency was also found for a few IQCs, which were found to be higher in 2008 than in any other year; after a change in IQCs in 2014, the performance was stable throughout all years, i.e. there was no significant difference in the MD_cut-off 7.0_ (ESM S2).

### Short-Term Experiment

All measurements were performed together with measurements taken for patient care under routine conditions. All results were complied using Rili-BAEK. Since the regular IQC samples were not part of the study design in the short-term approach, the %CVs from the routine IQC were taken separately: 2.6 and 1.9% at a glucose concentration of 5.2 and 14.7 mmol/L, respectively.

**Fig. 3:**
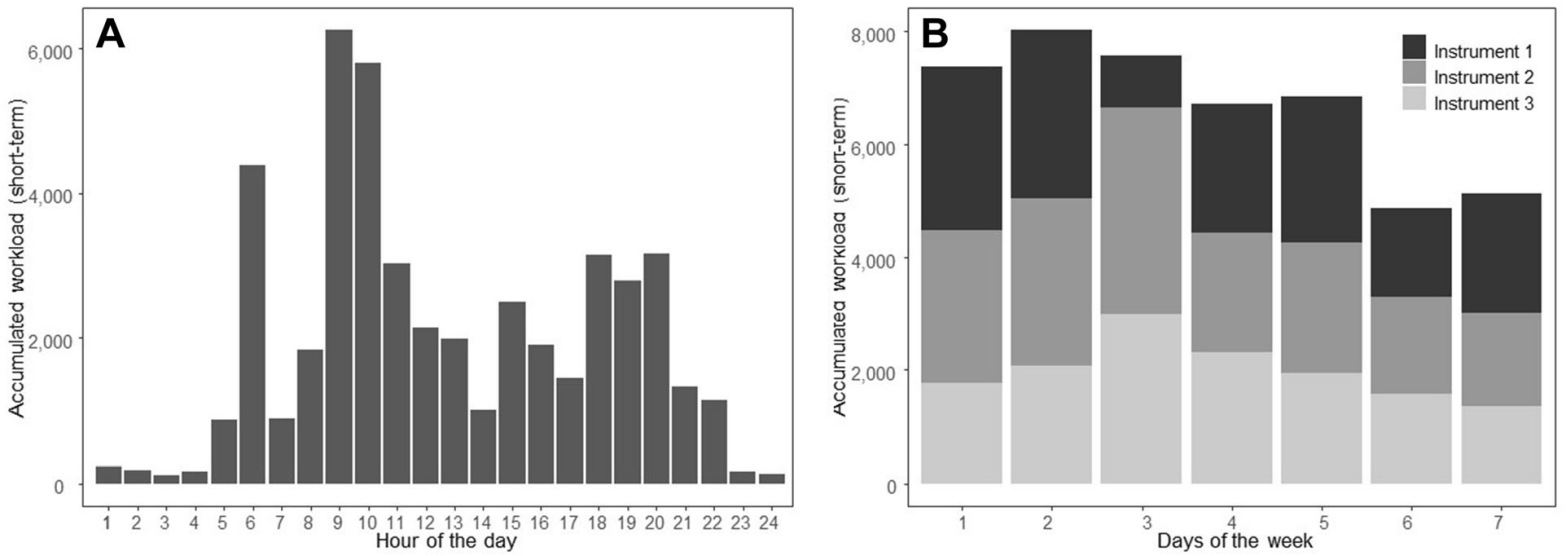
Workload of all instruments in number of tests according to hour of the day (**a**) and day of the week (**b**)

Evaluation of the bias among the instruments was possible since the sample material used in the short-term experiment was pooled and then distributed across the instruments. The bias of the median concentration ranged from − 0.1 to 0.1 mmol/L. Relative contributions of each instrument to the workload are given in Fig. 3.

#### Workload Profiles

A total of 1773 hourly glucose measurements for all concentrations was available for the analysis, which corresponds to 88% of all possible (*n* = 2016) measurements. The measurements that were missing were due to daily service and maintenance procedures. Examination of the daily workload profile, including all analytes provided for regular patient care on the Vista instruments (Fig. 3a), revealed one main peak at about 9–10 a.m., preceded by a smaller peak at 6 a.m., with a range of approximately 4000–6000 accumulated tests over the study period of 7 days. Between 500 and 800 laboratory tests were performed during these heavy workload hours. The lowest number of tests was recorded between 11 p.m and 4 a.m. The workload was shared approximately equally between the instruments, except for day 3 when two instruments took over most of the workload (Fig. 3b). Variations in results between and within days were noticeable but small (ESM S4) and showed no dependence on the workload.

#### Imprecision

The MD_cut-off_ of the three investigated glucose concentrations (3.3, 6.6 and 19.7 mmol/L) are summarized in Fig. 2. The linear regression (*y* = 0.052*x* + 0.032; Fig. 2d) of the three obtained MD_cut-off_ values enabled the calculation of the MD_cut-off 7.0_, which was 0.40 mmol/L.

The design of the short-term experiment permitted a more detailed evaluation, such as reporting daily variations within instruments for the investigated concentrations. In the short-term approach there was no significant difference between the MD_cut-off 7.0_ of the individual instruments or between days. The MD_cut-off 7.0_ of instrument 4 (Flexlab) was slightly but significantly higher than that of the other instruments. The MD_cut-off 7.0_ of the short-term experiment was comparable to that from the long-term experiment. The measurements results are summarized and reported in detail by day and instrument in ESM S4.

## Discussion

Clinicians should be aware of measurement uncertainty, such as, for example, when comparing the result of a glucose concentration measurement to a cut-off value used for the diagnosis of DM.

The MD_cut-off_ is a metric denoting the smallest analytical difference between a measurement and a cut-off that can be regarded as statistically significant at a 95% confidence level—provided a coverage factor (*k*) of 2 is applied [12]. Since the MD_cut-off_ is given in the unit of the analyte it can only be reported for a specific concentration. As the subject of our study was the MD_cut-off_ for the diagnosis of DM we focused on glucose concentrations at 7.0 mmol/L, which is the diagnostic cut-off value for FPG as recommended by ADA and DDG and assessed long-term and short-term imprecision expressed as MD_cut-off 7.0_ for glucose concentration measurements.

The MD_cut-off 7.0_ for glucose in the long-term and the short-term approaches were 0.44 and 0.40 mmol/L, respectively. The relation between the MD_cut-off_ and the glucose concentration represents the relative SD, i.e. the %CV. Any MD_cut-off_ on the linear regression line in Fig. 2 represents the identical %CV. A MD_cut-off_ above and below this regression line represents a higher and a lower %CV, respectively. In the laboratory setting, %CV can also be used to compare imprecisions of different quality control concentrations.

In our study, the MD_cut-off 7.0_ remained below the recommended limit of 0.7 mmol/L in both approaches. The results of the study illustrate the MD concept [6] as follows:

1. It can be expected that 5% of all glucose concentration measurements fall outside this MD_cut-off_. The central 95% interval of all measurement results in the short-term study was found to be 0.4–0.5 mmol/L at a glucose concentration of 6.6 mmol/L (ESM S4). This interval should resemble the MD_cut-off 7.0_ derived from the long-term experiment, which in turn was shown to be 0.44 mmol/L.
2. The differences between the highest and lowest glucose concentrations of study samples in the short-term experiment at 6.6 mmol/L varied between 0.6 and 0.7 mmol/L. Since a coverage factor *k* = 2 was chosen in this study, this result is reasonable. If the coverage factor (*k*) is increased to 3 to correspond to a 99% level of confidence, the MD_cut-off_ from the long-term study would be 0.67 mmol/L, which also matches our findings for minimum and maximum results in the short-term study.

In addition, routine Rili-BAEK IQC in the short-term approach, which relied on separate control material, confirmed that the %CV at a concentration of 6.6 mmol/L was 2.6%, which corresponds to a MD_cut-off_ of 0.5 mmol/L. This also resembles the central 95% interval of all measurement results in the short-term study.

The results of the present study are also in line with the MD_cut-off_ of 0.38 mmol/L at 5.0 mmol/L calculated from approximately 21,000 duplicate measurements of glucose in plasma collected from patients instead of from IQC material [13]. This result demonstrates that the control material used gives MD_cut-off_ results comparable to those determined for plasma samples from patients and therefore allows the MD_cut-off_ to be extrapolated to glucose concentration measurements of plasma samples from patients. Imprecision performance data claimed by the manufacturer are a SD = 0.12 mmol/L at 4.12 mmol/L and a SD = 0.46 mmol/L at 21.02 mmol/L, both of which can be used to derive a MD_cut-off 7.0_ of 0.36 (linear regression of *y* = 0.0402*x* + 0.0742) [14]. The imprecision given by the manufacturer is based on CLSI/National Committee for Clinical Laboratory Standard(NCCLS) EP5-A2: measurement of four samples of each concentration per day in two separate runs of two samples each for 20 days [4]. These imprecision results are lower than our findings indicating that imprecision based on the CLSI/NCCLS EP5-A2 protocol is not sufficient to provide a reliable MD_cut-off_.

The MD_cut-off 7.0_ were calculated from glucose concentration results obtained from three instruments run in parallel. The MD_cut-off_ for individual instruments differed significantly in the long-term approach, but not in the short-term approach, indicating that a longer period of time is needed for an assessment of a reliable MD_cut-off 7.0_. Furthermore, the MD_cut-off_ for the individual instruments were slightly lower than those from the overall system; this result was expected since in addition to the larger imprecision, slight biases between the instruments also occurred, thereby adding to the variation when examining the overall distribution system ‘instrument 4.’

The results reported to clinicians, however, represent the combined performance of all instruments. The results of this study show that even when each instrument is shown to have a good assay performance, the clinician would experience differences in glucose results as large as 1.0 mmol/L at a glucose concentration of 6.6 mmol/L (minimum 6.2 mmol/L; maximum 7.2 mmol/L) due to imprecision and bias among the instruments. Therefore, the MD_cut-off_ calculation should be based on MD distribution from all instruments that are connected to the automated distribution system.

Data from the long-term study show a reliable stability of the investigated systems over one decade, demonstrating the high quality of the manufacturer and medical laboratories. The effectiveness of the quality assurance systems, such as Rili-BAEK, with internal and external quality controls also contribute to the stable performance over time.

Short-term data showed minor performance shifts within the instruments, but these could not be linked to shifts in the workload. Even though the systems were observed to have a high stability, it has to be noted that the minimum QC frequency by Rili-BAEK allowed hundreds of patient results to be released in-between IQCs. An increase IQC frequency would reduce the number of released patient results, but it cannot be excluded that incidences between two IQC results may still occur unnoticed. Still, a higher IQC frequency reduces the number of samples that need to be retested after a failed IQC. It is up to the individual medical laboratory to carefully balance costs and quality of patient care.

The variability introduced by slight differences in performance within and between instruments can be covered by reporting the long-term MD_cut-off_ across all connected instruments. Stable results for MD_cut-off 7.0_ were obtained after about 30 independent control cycles of MD_cut-off 7.0_, a number that was reached after 1 year of combining MD_cut-off 7.0_ data from all three instruments.

The between-year MD_cut-off 7.0_ values were not significantly different, except for the first year of the long-term approach when the instruments had just been introduced to the laboratory. Thus, medical laboratories may use the IQC of about 1 year to provide a reliable MD_cut-off_ to report along with glucose concentration results used to diagnose DM. This finding becomes especially important when close diagnostic cut-offs increase the need for reliable results to classify individuals correctly [15]. Subsequently, continuous monitoring of the MD_cut-off_ builds an even more reliable database and can also facilitate identification of performance changes as well as comparisons of different measurement methods and laboratories.

### Limitations

The study was limited to one type of instrument (Dimension Vista 1500), and the short-term imprecision part was limited to 1 week only. Adverse effects may have occurred less often than weekly and, therefore, these could not be identified in the short-term approach study due to its design. The commercially available IQC material used in the study closely resembles patient material, but is not patient material; therefore, effects due to any differences cannot be completely excluded. The aim of the study design was to assess measurement imprecision and express this imprecision as the MD.

## Conclusion

Imprecision for glucose concentration measurements, assessed as MD_cut-off 7.0_ from monthly IQC control cycles over a period of 10 years, was 0.44 mmol/L and, therefore, well below the recommended limit of 0.7 mmol/L. Hourly measurements made over a 1-week period confirmed these findings and illustrated the MD concept. Imprecision as measured by IQC is also remarkably stable over many years of operation.

Current imprecision assessment focuses only on single instruments, whereas clinicians perceive the combined analytical performance of all instruments used for a certain assay in a given laboratory. Therefore, we suggest deriving the MD_cut-off_ from all instruments and control cycles that are used in the setting of patient care in a given medical laboratory. In our study, about 30 independent control cycles provided sufficient data to determine a reliable MD_cut-off_. Establishing a continuous monitoring of MD_cut-off_ may complement traditional quality assurance.

## Electronic supplementary material

Below is the link to the electronic supplementary material.

Supplementary material 1 (PDF 564 kb)
