# Supplementary material for: Measurement Uncertainty Impacts Diagnosis of Diabetes Mellitus: Reliable Minimal Difference of Plasma Glucose Results
Source: Diabetes Ther. 2019 Dec 16;11(1):293–303. doi: 10.1007/s13300-019-00740-w (PMC6965559; doi:10.1007/s13300-019-00740-w)
Supplement: Supplementary file 1 — Supplementary material 1 (PDF 564 kb) [file 13300_2019_740_MOESM1_ESM.pdf]

Supplement S 3: Distribution of $MD_{cut\text{-}off}$ at the concentrations of the applied control material for the individual instruments during the study period of ten years. The 95th percentile $MD_{cut\text{-}off}$ values are given above the boxplots.

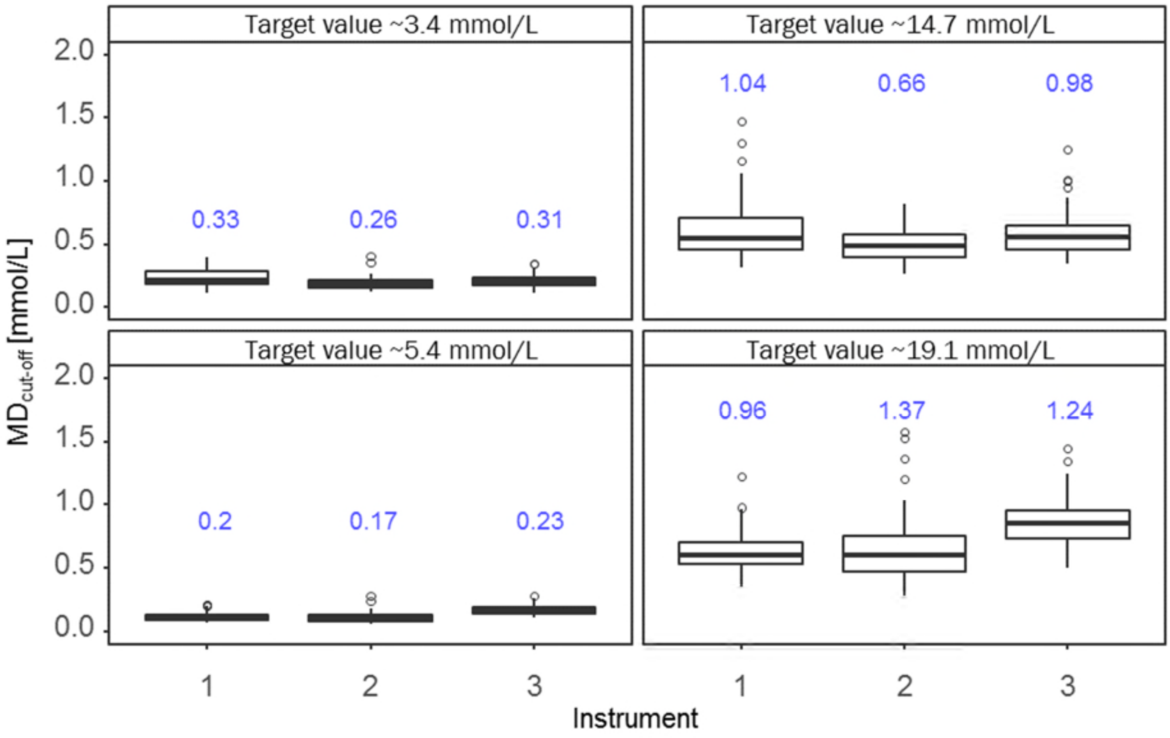

Supplement S 4: Results for glucose displayed by instruments aggregated over the study period. Horizontal solid lines represent target values, dashed lines represent Rili-BAEK-limits and horizontal dotted lines represent maximum $MD_{cut\text{-}off\ 7.0}$.

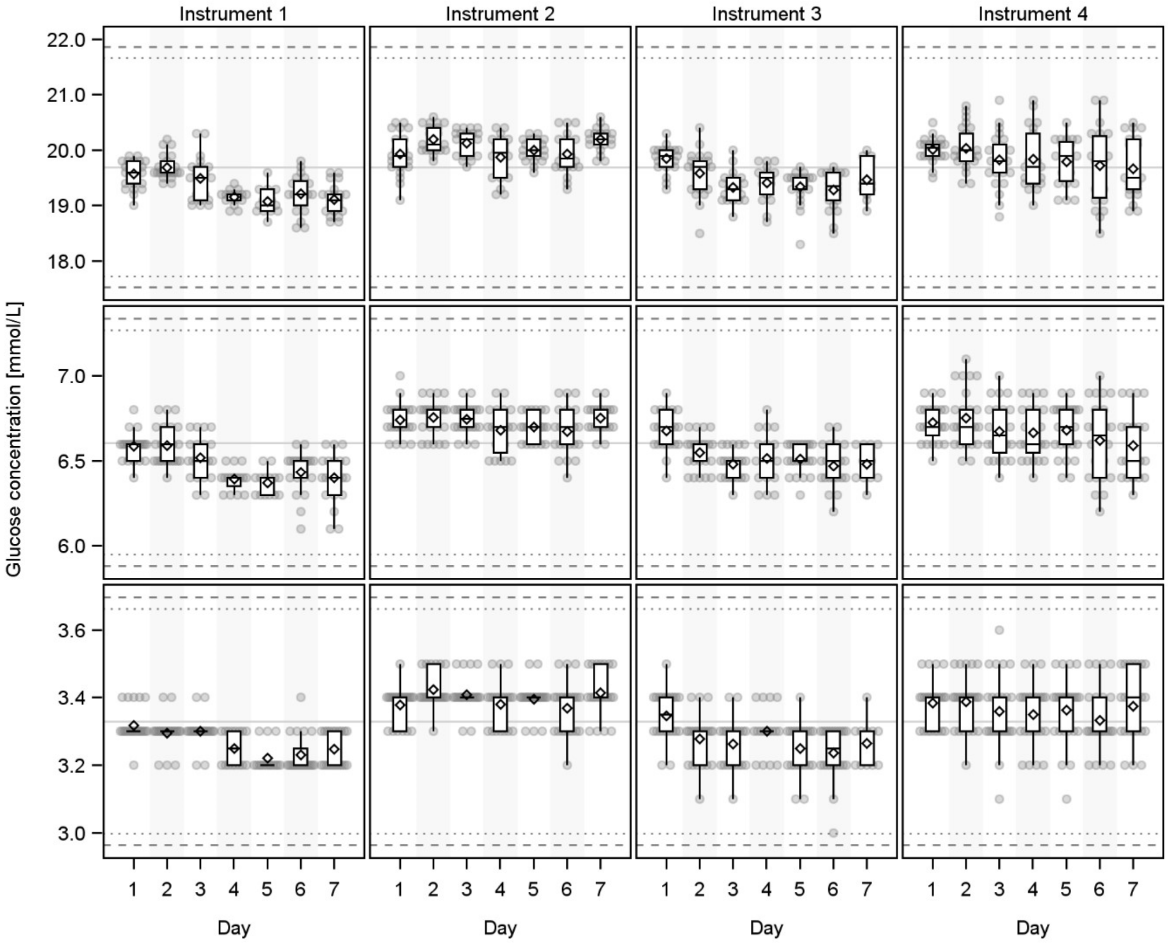

```
########## Suppl. S1 - "Measurement uncertainty impacts diagnosis of diabetes mellitus: reliable minimal difference of plasma glucose results",
# by Sandra Keutmann, Stephanie Zylla, Mathilde Dahl, Nele Friedrich, Rüdiger Landgraf, Lutz Heinemann, Anders Kallner, Matthias Nauck, Astrid Petersmann
###########################################
library(readxl)
library(ggplot2)
require(plyr)
library(export)
library(qdapTools) # lookup
library(tidyverse)
library(okcupiddata)
library(reshape)
library(dplyr)
library(data.table)
library(rlang)
library(agricolae) # HSD test
library(broom)


####################################################################################################
####
####                   Long term study
####
####################################################################################################


### Data ######
mdall<- read_excel("qk_gluk_gesamt.xlsx", sheet=1,col_names=T)
mdall <- dplyr::filter(mdall, KONTRSERUMID == "MU1" | KONTRSERUMID == "MU3" | KONTRSERUMID == "M1" | KONTRSERUMID == "M2")
mdall <- filter(mdall, GERAET == "Caesar (SN 367)" | GERAET == "Dieter (SN 385)"| GERAET == "Ede (SN 541)")


### table for mashine (geraet), QC level (KONTRSERUMID), month and CHARGENNR
table <- ddply(mdall, c("GERAET", 'KONTRSERUMID', 'MONAT','JAHR','CHARGENNR'), summarise,
               median = median(ERGEBNISF),
               mean = mean(ERGEBNISF),
               SD = sd(ERGEBNISF),
               MD = SD*2, ### only when comparing to ref material !
               mean_ref_SOLLWERT = mean(SOLLWERT),
               sd_ref_SOLLWERT = sd(SOLLWERT),
               MD_pct = MD/mean_ref_SOLLWERT*100,
               N = length(ERGEBNISF),
               Q05 = round(quantile(ERGEBNISF, .05), digits = 2),
               Q95 = round(quantile(ERGEBNISF, .95), digits = 2)
               )

#### Remove outliers
```

```
table <- table[c(-176,-453),]

### Only those with > 14 measurments per MD cycle
table <- table[which(table$N > 14),]


boxplot_table_targetvalues <- ddply(table, c('KONTRSERUMID'), summarise,
                       median = median(MD),
                       mean = mean(MD),
                       N = length(MD),
                       target = mean(mean_ref_SOLLWERT),
                       Q05 = round(quantile(MD, .05), digits = 2),
                       Q95 = round(quantile(MD, .95), digits = 2)
                       )

boxplot_table_mashine <- ddply(table, c('GERAET','KONTRSERUMID'), summarise,
                                    median = median(MD),
                                    mean = mean(MD),
                                    N = length(MD),
                                    Q05 = round(quantile(MD, .05), digits = 2),
                                    Q95 = round(quantile(MD, .95), digits = 2)
                               )

sum_N <- ddply(table, c('KONTRSERUMID'), summarise,
               sum = sum(N),
               mean = mean(mean_ref_SOLLWERT)
               )

sum_percent <- ddply(table, c( 'KONTRSERUMID'), summarise,
                     MD_pct_av = mean(MD_pct),
                     sd_pct_av = sd(MD_pct)
                     )

sum_percent_mashine <- ddply(table, c( 'GERAET','KONTRSERUMID'), summarise,
                             MD_pct_av = mean(MD_pct),
                             sd_pct_av = sd(MD_pct)
                             )


table_MU1_M3 <- table[, c('KONTRSERUMID', 'MONAT','JAHR')]
table_MU1_M3 <-unique(table_MU1_M3)

#### TWO-way ANOVA
table$combi <- paste(table$GERAET, table$JAHR)
MU1 <- table[which(table$KONTRSERUMID == 'MU1'),]
MU3 <- table[which(table$KONTRSERUMID == 'MU3'),]
M1 <- table[which(table$KONTRSERUMID == 'M1'),]
M2 <- table[which(table$KONTRSERUMID == 'M2'),]
material_list <- list(M1, M2, MU1, MU3)
summary(material_list)

name_list <- c('M1', 'M2', 'MU1', 'MU3')

for(i in 1:length(material_list)){
```

```
  dat <- as.data.frame(material_list[i])
  fit <- lm(MD_pct ~ GERAET , data = dat) # GERAET or JAHR
  aov1 <- aov(fit)
  print(summary(aov1) )
  # Tukey Honestly Significant Differences
  group_test <- HSD.test(aov1, 'GERAET')  # GERAET or JAHR
  print(group_test)

}

  fit <- lm(MD_pct ~ GERAET , data = tabel) # GERAET or JAHR
  aov1 <- aov(fit)
  print(summary(aov1) )
  # Tukey Honestly Significant Differences
  group_test <- HSD.test(aov1, 'GERAET')  # GERAET or JAHR
  print(group_test)



###################################
### PLOTTING
###################################
 plot <- table
 plot2 <- boxplot_table_targetvalues[,c(5,7)]

#### Per machine
################

p <- ggplot(plot, aes(x = GERAET, y = MD))+
  geom_boxplot(outlier.shape = 1)+#ggtitle(label = "MDcut-off QC material long 
2009-2018")+
 theme_bw()+ theme(panel.grid = element_blank())+ ylim(0,2)+
  scale_x_discrete(labels=c("1", "2", "3"))+ xlab('Measuring system')+
  ylab('MDcut-off [mmol/L]')+ facet_wrap(.~KONTRSERUMID)
p


####### Old and new controles
#############################

plot$KONTRSERUMID_sort <- factor(plot$KONTRSERUMID, levels = c("M1","MU1","M2", 
"MU3"))
plot$KONTRSERUMID <- as.character(plot$KONTRSERUMID)

#### SUPER smart lookup!!!
ave <- sum_N[,c(1,3)]
categories = round(ave$mean,1)
names(categories) = ave$KONTRSERUMID

plot$lab = categories[plot$KONTRSERUMID]
unique(plot$lab) #  5.4 14.7  3.4 19.1
plot$lab <- factor(plot$lab, levels = c("3.4","5.4","14.7","19.1"))
```

```
p2 <- ggplot(plot, aes(x = lab, y = MD))+
  geom_boxplot(outlier.shape =1)+ ggtitle(label = "MDcut-off QC material long
2009-2018")+
  theme_bw()+ theme(panel.grid = element_blank())+
  ylim(0,2) + xlab('Target value')+ ylab('MDcut-off [mmol/L]')
p2


### adding N in the plot

meds <- c(8,8,8,8)
names(meds) <- c('M1', 'M2', 'MU1', 'MU3')

p3 <- ggplot(plot, aes(x = KONTRSERUMID, y = MD_pct))+
  geom_boxplot()+ ggtitle(label = "MD for QC material", subtitle = "01.01.2009 -
31.12.2018")+
  theme(axis.text.x= element_text(angle=45, hjust = 1.3, vjust = 1.2))+
xlab('')+ylab('MD[%]')
p3 <- p3 +geom_text(data=data.frame(), aes(x=names(meds), y=meds,
label=as.character(sum_N$sum)), col='blue', size=4,
            vjust = 1)
p3

#### Per cent
p4 <- ggplot(plot, aes(x = lab, y = MD_pct))+
  geom_boxplot(outlier.shape =1)+ #ggtitle(label = "MD for QC material",
subtitle = "01.01.2009 - 31.12.2018")+
  theme_bw()+  theme(axis.text.x= element_text(angle=45, hjust = 1.3, vjust =
1.2),panel.grid = element_blank())+ xlab('')+ylab('MD[%]')+ ylim(0,8)
p4 #<- p4+geom_text(data=data.frame(), aes(x=names(meds), y=meds,
label=as.character(sum_N$sum)), col='blue', size=4, vjust = 15)
p4


### Linear regression

p5 <- ggplot(plot2, aes(x = target, y = Q95))+ geom_point()+
  geom_smooth(method='lm') + geom_hline(yintercept =0.442) +
geom_vline(xintercept =7.0)+theme_bw()
p5

## adding the lines for 7.0 mmol/L
###################################
# Equation see below
#0.065*x -0.013 = y
#x = 7 ; (0.065*7)-0.013 = 0.442 = y

x <- 7.0
y <- 0.442
# then you can use:
plot(x = plot2$target, y = plot2$Q95, pch = 1, cex=1.5,xaxt='n', xlab = 'Glucose
concentration [mmol/L]', ylab = 'MD cut-off [mmol/L]')
axis(side = 1, at=c(5,7,10,15))
lines(x=c(x,x), y=c(0,y), lwd=1,lty = 2)
```

```
lines(x=c(0,x), y=c(y,y), lwd=1, lty = 2)
lm(plot2$Q95 ~ plot2$target)
abline(-0.01338, 0.06525,lwd=1, col= 'blue')
abline(h=0.70,lwd=1,lty = 2 )
### get the equation

lm_eqn <- function(df){
  m <- lm(y ~ x, df);
  eq <- substitute(italic(y) == a + b %.% italic(x)*","~~italic(r)^2~"="~r2,
                   list(a = format(coef(m)[1], digits = 2),
                        b = format(coef(m)[2], digits = 2),
                        r2 = format(summary(m)$r.squared, digits = 3)))
  as.character(as.expression(eq));
}

stat <- plot2
names(stat) <- c('x', 'y')
p5 <- p5 + geom_text(x = 10, y = 0.5, label = lm_eqn(stat), parse = TRUE)
p5


### Export to PowerPoint
####################################################################
################################################################################
#############
graph2ppt(x= p ,file="MDlong.pptx", width=8, height=5)
graph2ppt(x= p2 ,file="MDlong.pptx", width=8, height=5, append=T)
graph2ppt(x= p3 ,file="MDlong.pptx", width=8, height=5, append=T)
graph2ppt(x= p4 ,file="MDlong.pptx", width=8, height=5, append=T)



################################################################################
###################
####
####                   Short term study
####
################################################################################
###################

##### DATA
md.short <- read_excel("2019 Mar 31 short term data glucose.xlsx",
sheet=1,col_names=T)
md.short.clean <- na.omit(md.short)

#5 and 95% percentile system4 medium 6.6 mmol/L
md.short.clean.med.sys4 <- filter(md.short.clean, SYSTEM=="flexlab",
QC._LEVEL=="med" )
round(quantile(md.short.clean.med.sys4$GLUK, .05), digits = 2)
round(quantile(md.short.clean.med.sys4$GLUK, .95), digits = 2)
round(median(md.short.clean.med.sys4$GLUK, na.rm=TRUE), digits = 2)

#5 and 95% percentile system1 medium 6.6 mmol/L
md.short.clean.med.sys1 <- filter(md.short.clean, SYSTEM=="caesar", QC._LEVEL ==
```

```
"med" )
round(quantile(md.short.clean.med.sys1$GLUK, .05), digits = 2)
round(quantile(md.short.clean.med.sys1$GLUK, .95), digits = 2)
round(median(md.short.clean.med.sys1$GLUK, na.rm=TRUE), digits = 2)

#5 and 95% percentile system2 medium 6.6 mmol/L
md.short.clean.med.sys2 <- filter(md.short.clean, SYSTEM=="dieter", QC._LEVEL ==
"med" )
round(quantile(md.short.clean.med.sys2$GLUK, .05), digits = 2)
round(quantile(md.short.clean.med.sys2$GLUK, .95), digits = 2)
round(median(md.short.clean.med.sys2$GLUK, na.rm=TRUE), digits = 2)

#5 and 95% percentile system3 medium 6.6 mmol/L
md.short.clean.med.sys3 <- filter(md.short.clean, SYSTEM=="ede", QC._LEVEL ==
"med" )
round(quantile(md.short.clean.med.sys3$GLUK, .05), digits = 2)
round(quantile(md.short.clean.med.sys3$GLUK, .95), digits = 2)
round(median(md.short.clean.med.sys3$GLUK, na.rm=TRUE), digits = 2)

#qc cycles with n and SD
md.short.clean.grouped <- group_by(md.short.clean, SYSTEM, DAY, QC._LEVEL)
md.short.clean.grouped.sd <- summarise(md.short.clean.grouped, n_per_cycle =
n(), sd_cycle = sd(GLUK))
summary(md.short.clean.grouped.sd)
#qc cycles md
md.short.clean.grouped.md  <- mutate(md.short.clean.grouped.sd, md_cycle =
sd_cycle*2)
summary(md.short.clean.grouped.md)

#Anzahl pro Kontrollzyklus eingrenzen, Ausschluss geringe pro Kontrollzyklus
bb <- filter(md.short.clean.grouped.md, n_per_cycle >14)
summary(bb)
ggplot(bb, aes(DAY, QC._LEVEL)) + geom_point()

#subsetting per QC level
summary(bb)
bb.low <- filter(bb, QC._LEVEL == "low")
bb.med <- filter(bb, QC._LEVEL == "med")
bb.upp <- filter(bb, QC._LEVEL == "upp")
boxplot(bb.low$md_cycle, bb.med$md_cycle, bb.upp$md_cycle, las=2, ylab="MD
(cut-off) [mmol/L]", ylim=c(0,2))

bb_all <- rbind(bb.low, bb.med)
bb_all <- rbind(bb_all, bb.upp)

names(bb_all)

cat = c("3.3", "6.6", "19.7")
names(cat) = c("low", "med", "upp")

md.short.clean.grouped$lab = cat[md.short.clean.grouped$QC._LEVEL]
md.short.clean.grouped$lab <- as.numeric(md.short.clean.grouped$lab)

### table for mashine (geraet), QC level (KONTRSERUMID), month and CHARGENN
```

```
table_shot <- ddply(md.short.clean.grouped, c( "SYSTEM","DAY", "QC._LEVEL"),
summarise,
                    median = median(GLUK),
                    n_per_cycle = n(),
                    mean = mean(GLUK),
                    SD = sd(GLUK),
                    MD = SD*2, ### only when comparing to ref material !
                    mean_ref = mean(lab),
                    sd_ref = sd(lab),
                    MD_pct = MD/mean_ref*100,
                    N = length(GLUK),
)

### ONly those with more than 14 cycles per calculation
table_shot_red <- filter(table_shot, n_per_cycle >14)

boxplot_table_targetvalues <- ddply(table_shot_red, c('QC._LEVEL'), summarise,
                                    median = median(MD),
                                    mean = mean(MD),
                                    N = length(MD),
                                    target = mean(mean_ref),
                                    Q05 = round(quantile(MD, .05), digits = 2),
                                    Q95 = round(quantile(MD, .95), digits = 2)
                                    )


sum_percent_short <- ddply(table_shot_red, c( 'QC._LEVEL'), summarise,
                           MD_pct_av = mean(MD_pct),
                           sd_pct_av = sd(MD_pct)
)


p <- ggplot(bb_all, aes(x = QC._LEVEL, y = md_cycle))+
  geom_boxplot(outlier.shape =1)+
  theme_bw()+ theme(panel.grid = element_blank())+
  ylim(0,2) + xlab('')+ ylab('MD [mmol/L]')+ggtitle('MDcut-off QC material short
term')
p


### Regression plot
tab <- as.data.frame(c('3.3'=round(quantile(bb.low$md_cycle, .95), digits =
3),'6.6'=round(quantile(bb.med$md_cycle, .95), digits =
2),'19.7'=round(quantile(bb.upp$md_cycle, .95), digits = 2))
)
names(tab) <- 'MD_Q95_cut'
tab$target <- row.names(tab)
tab$target <- gsub('.95%', '', tab$target)
tab$target <- as.numeric(tab$target)

## get the equation

lm_eqn <- function(df){
  m <- lm(y ~ x, df);
```

```
  eq <- substitute(italic(y) == a + b %.% italic(x)*","~~italic(r)^2~"="~r2,
                   list(a = format(coef(m)[1], digits = 2),
                        b = format(coef(m)[2], digits = 2),
                        r2 = format(summary(m)$r.squared, digits = 3)))
  as.character(as.expression(eq));
}

names(tab) <- c('y', 'x')
lm_eqn(tab)

# Equation see below
#0.052*x 0.032 = y
#x = 7 ; (0.052*7)+0.032 = 0.396 = y

x <- 7.0
y <- 0.396
# then you can use:
plot(x = tab$x, y = tab$y, pch = 1, cex=1.5,xaxt='n', xlab = 'Glucose
concentration [mmol/L]', ylab = 'MD cut-off [mmol/L]')
axis(side = 1, at=c(5,7,10,15))
lines(x=c(x,x), y=c(0,y), lwd=1,lty = 2)
lines(x=c(0,x), y=c(y,y), lwd=1, lty = 2)
lm(tab$y ~ tab$x)
abline( 0.03222 ,0.05163,lwd=1, col= 'blue')
abline(h=0.70,lwd=1,lty = 2 )


#### TWO-way ANOVA
low <- table_shot_red[which(table_shot_red$QC._LEVEL == 'low'),]
med <- table_shot_red[which(table_shot_red$QC._LEVEL == 'med'),]
upp <- table_shot_red[which(table_shot_red$QC._LEVEL == 'upp'),]

ede <- table_shot_red[which(table_shot_red$SYSTEM == 'ede'),]
dieter <-table_shot_red[which(table_shot_red$SYSTEM == 'dieter'),]
caecar<-table_shot_red[which(table_shot_red$SYSTEM == 'caesar'),]
sys4<-table_shot_red[which(table_shot_red$SYSTEM == 'flexlab'),]

material_list <- list(low, med, upp)

material_list <- list(ede, dieter, caecar, sys4)
summary(material_list)


for(i in 1:length(material_list)){
  dat <- as.data.frame(material_list[i])
  fit <- lm(MD_pct ~ DAY , data = dat) # GERAET or JAHR or DAY
  aov1 <- aov(fit)
  print(summary(aov1) )
  # Tukey Honestly Significant Differences
  group_test <- HSD.test(aov1, 'DAY')  # GERAET or JAHR or DAY
  print(group_test)

}
head(table_shot_red)
```

```
table_shot_red$combi <- paste(table_shot_red$SYSTEM, table_shot_red$QC._LEVEL,
sep = '_')

fit <- lm(MD_pct ~ combi , data = table_shot_red) # GERAET or JAHR or SYSTEM
aov1 <- aov(fit)
print(summary(aov1) )
# Tukey Honestly Significant Differences
group_test <- HSD.test(aov1, 'combi')  # GERAET or JAHR or SYSTEM
print(group_test)

################################################
##### Work load plots

work <- read.csv('IQC_Studie_Probenzahl.csv')
dates <- strsplit(as.character(work$DATUM), split="/")
work$day<-sapply(dates, function(x) paste(x[1]))
work$month <-sapply(dates, function(x) paste(x[2]))

work <- work[which(work$month=='09'),]
work$day <- as.numeric(work$day)
work <- work[which(work$day >14 & work$day<22),]

p <- ggplot(work, aes(x = ZEITBER, y = ANZAHL))+
  geom_bar(stat="identity")+
  theme_bw()+ theme(panel.grid = element_blank())+
  xlab('Hour of the day')+ ylab('Accumulated
workload')+ggtitle('')+scale_x_continuous(limits=c(0, 25), breaks=c(0:25,1))
p


p <- ggplot(work, aes(x = WOCHENTAG, y = ANZAHL))+
  geom_bar(aes(fill = GERAET), stat="identity")+
  theme_bw()+ theme(panel.grid = element_blank())+  scale_fill_grey()+
  xlab('Day of week')+ ylab('Accumulated workload')+ggtitle('')
+scale_x_discrete(
limits=c('Montag','Dienstag','Mittwoch','Donnerstag','Freitag','Samstag',
'Sonntag'))
p
```

Supplement S2

Analysis of variance between instruments (I) and materials (II) across years/days (III) for long term (A) and short term (B).

A)

(I)

**Instrument**	**%MD**	**Group (P< 0.05)**
3	4.6	a
2	3.9	ab
1	3.5	b

(II)

**Target 3.4**	**%MD**	**Group (P< 0.05)**
1	4.2	a
3	3.9	ab
2	3.6	b
**Target 14.7**		
1	4.1	a
3	4.0	a
2	3.3	b
**Target 5.4**		
3	5.0	a
1	3.5	b
2	3.3	b
**Target 19.1**		
3	4.6	a
2	3.9	ab
1	3.5	b

(III)

**Instrument**	Year	**T3.4 (%MD)**	groups (p<0.05)	**T14.7 (%MD)**	groups (p<0.05)
1	2008	5.4	a	6.9	a
1	2009	4.3	ab	4.2	b
1	2010	4.5	ab	4.0	b
1	2011	4.3	ab	4.1	b
1	2012	3.9	ab	3.3	b
1	2013	3.6	b	3.3	b
2	2009	4.4	a	3.7	a
2	2010	3.5	a	3.8	a
2	2011	3.7	a	3.6	a
2	2012	3.4	a	2.8	a
2	2013	3.2	a	3.0	a
3	2010	4.5	a	4.8	a
3	2011	3.6	a	3.6	a
3	2012	3.7	a	3.4	a

3	2013	3.9	a	4.3	a

**Instrument**	Year	**T5.4 (%MD)**	groups (p<0.05)	**T19.1 (%MD)**	groups (p<0.05)
1	2014	4.4	a	3.8	a
1	2015	3.4	a	3.2	a
1	2016	3.3	a	3.5	a
1	2017	2.9	a	3.7	a
1	2018	3.2	a	3.1	a
2	2014	2.7	a	2.7	b
2	2015	2.6	a	2.6	b
2	2016	3.4	a	3.8	ab
2	2017	3.6	a	6.2	a
2	2018	4.6	a	4.3	ab
3	2014	4.8	a	4.8	a
3	2015	5.5	a	4.4	a
3	2016	4.7	a	4.3	a
3	2017	5.0	a	4.6	a
3	2018	5.0	a	4.7	a

B)

(I)

Instrument	%MD	Group (P< 0.05)
Flexlab	5.08	a
Instrument 3	3.72	b
Instrument 2	3.27	b
Instrument 1	3.00	b

(II)

Target 3.3	%MD	Group (P< 0.05)
Instrument 1	3.08	c
Instrument 2	3.82	bc
Instrument 3	4.51	abc
Flexlab	5.65	a
**Target 6.6**		
Instrument 1	3.28	a
Instrument 2	3.03	a
Instrument 3	3.43	ab
Flexlab	4.99	b
**Target 19.7**		
Instrument 1	2.68	a
Instrument 2	2.96	a
Instrument 3	3.20	ab
Flexlab	4.61	b

(III)

Instrument 3	%MD	Group (P< 0.05)
Day 1	3.44	a
2	3.97	a
3	3.29	a
4	4.13	a
5	3.42	a
6	4.04	a
**Instrument 2**		
Day 0	3.62	a
1	3.35	a
2	2.87	a
3	2.70	a
4	4.07	a
5	2.40	a
6	4.15	a
7	3.00	a
**Instrument 1**		
Day 0	3.17	a
1	2.70	a
2	2.83	a

3	3.49	a
4	2.19	a
5	2.41	a
6	3.59	a
7	3.44	a
**Flexlab**		
Day 1	3.22	a
2	4.56	ab
3	5.45	ab
4	5.19	ab
5	4.91	ab
6	6.44	b
7	5.78	b
